# Symptomatic generalised joint hypermobility and autism spectrum disorder are associated in adults

**DOI:** 10.1192/j.eurpsy.2022.1145

**Published:** 2022-09-01

**Authors:** M. Glans, S. Bejerot, M. Elwin, M. Humble

**Affiliations:** 1 Faculty of Medicine and Health, University Health Care Research Centre, Örebro, Sweden; 2 Örebro University, Faculty Of Medicine And Health, Örebro, Sweden

**Keywords:** biomarkers, comordbidity, hypermobility, Autism Spectrum Disorder

## Abstract

**Introduction:**

Intriguingly, autism spectrum disorders (ASD) and symptomatic generalised joint hypermobility (S-GJH) (e.g. hypermobility spectrum disorders and Ehlers Danlos Syndrome) share several clinical manifestations including motor difficulties, sensory hypersensitivity and autonomic dysfunction. Moreover, many syndromic forms of ASD manifest a hypermobile phenotype. Despite the increased interest in the area, few systematic studies are available.

**Objectives:**

This large cross-sectional comparative study aimed to examine the association between S-GJH and ASD in adults.

**Methods:**

We assessed GJH by physical examination using the Beighton Scoring System (BSS) and collected data on musculoskeletal symptoms and skin abnormalities amongst 156 adult patients with ASD and 413 adult community controls. A proxy for S-GJH was created by combining a positive BSS with at least one additional musculoskeletal symptom or skin abnormality.

**Results:**

The prevalence of S-GJH was significantly higher amongst patients with ASD than amongst controls (16.7% vs 4.8%, p< .001). A logistic regression model, adjusting for candidate covariates of GJH (age, sex, race), revealed a significant influence of ASD on S-GJH with adjusted odds ratio of 5.4 (95% CI 2.8-10.5, p< .001).

**Conclusions:**

ASD and S-GJH are associated in adults. If recognised, musculoskeletal complications related to S-GJH can be relieved by physiotherapy. Clinicians should be familiar with that symptoms frequently occurring in GJH such as pain, fatigue and orthostatic intolerance may mimic or aggravate psychiatric symptoms (e.g. depression, anxiety). Knowledge about comorbidities may provide clues to underlying aethiopathological factors. Future research to clarify the mechanisms behind this association and to evaluate how comorbid S-GJH affects ASD outcome is warranted.

**Disclosure:**

No significant relationships.

